# Long-Term Nitrogen Dioxide Exposure as a Possible 5-Year Mortality Risk Factor in Diabetic Patients Treated Using Off-Pump Surgical Revascularization—A Retrospective Analysis

**DOI:** 10.3390/medicina60081326

**Published:** 2024-08-15

**Authors:** Tomasz Urbanowicz, Krzysztof Skotak, Aleksandra Krasińska-Płachta, Mariusz Kowalewski, Anna Olasińska-Wiśniewska, Krystian Szczepański, Andrzej Tykarski, Beata Krasińska, Zbigniew Krasiński, Marek Jemielity

**Affiliations:** 1 Cardiac Surgery and Transplantology Department, Poznan University of Medical Sciences, 61-848 Poznan, Poland; tomasz.urbanowicz@skpp.edu.pl (T.U.);; 2 Thoracic Research Centre, Collegium Medicum Nicolaus Copernicus University, Innovative Medical Forum, 85-094 Bydgoszcz, Poland; 3 Institute of Environmental Protection—National Research Institute, 02-170 Warsaw, Poland; 4 Department of Ophthalmology, Poznan University of Medical Sciences, 60-569 Poznan, Poland; 5 Department of Cardiac Surgery and Transplantology, National Medical Institute of the Ministry of Interior and Administration, 02-507 Warsaw, Poland; 6 Cardio-Thoracic Surgery Department, Heart and Vascular Centre, Maastricht University Medical Centre (MUMC), Cardiovascular Research Centre Maastricht (CARIM), 6229 HX Maastricht, The Netherlands; 7 Department of Hypertensiology, Angiology and Internal Medicine, Poznan University of Medical Sciences, 61-848 Poznan, Poland; 8 Department of Vascular, Endovascular Surgery, Angiology and Phlebology, Poznan University of Medical Science, 61-848 Poznan, Poland

**Keywords:** NO_2_, nitrogen dioxide, diabetes mellitus, air pollution, OPCAB

## Abstract

*Background*: There is mounting evidence that diabetic-related cardiac metabolism abnormalities with oxidative stress and inflammatory mechanism activation align with the functional impairments that result in atherosclerotic lesion formation. Among the possible non-traditional coronary lesion risk factors, environmental exposure may be significant, especially in diabetic patients. *Methods*: A total of 140 diabetic patients (115 (82%) males and 25 (18%) females) with a mean age of 65 (60–71) underwent surgical revascularization due to multivessel coronary disease. The possible all-cause mortality risk factors, including demographical and clinical factors followed by chronic air pollution exposure, were identified. *Results*: All patients were operated on using the off-pump technique and followed for 5.6 (5–6.1) years. The multivariable model for 5-year mortality prediction presented the nitrogen dioxide chronic exposure (HR: 3.99, 95% CI: 1.16–13.71, *p* = 0.028) and completeness of revascularization (HR: 0.19, 95% CI: 0.04–0.86, *p* = 0.031) as significant all-cause mortality risk factors. *Conclusions*: Ambient air pollutants such as an excessive chronic nitrogen dioxide concentration (>15 µg/m^3^) may increase 5-year all-cause mortality in diabetic patients following surgical revascularization.

## 1. Introduction

The current trend of an increasing population age is giving rise to the co-existence of coronary artery disease. As the intricacies of cardiovascular disease (CVD) unfold, identifying proper risk factors is of utmost importance. Multivessel coronary artery disease represents the advanced epicardial atherosclerotic involvement that requires intervention to improve patients’ life expectancy. Percutaneous interventions and the surgical approach are the optimal therapeutic approaches that improve long-term life expectancy [1].

The growing prevalence of diabetes mellitus has been observed in recent years [2], encompassing atherosclerosis risk. Chronic exposure to hyperglycemia leads to multiorgan dysfunction through interference with microvasculopathic degeneration and possesses a high impact on overall life expectancy.

There is mounting evidence that diabetic-related cardiac metabolism abnormalities with oxidative stress and inflammatory mechanism activation align with the functional impairments that result in atherosclerotic lesions formation [3]. The genetic background of diabetes and epicardial atherosclerosis represent different genetic architectures but are characterized by pleiotropy [4]. Patients presenting with chronic coronary syndrome and concomitant diseases such as diabetes mellitus are believed to benefit most from surgical revascularization [5]. More importantly, the clinically hidden earlier involvement of coronary arteries by atherosclerosis lesions is postulated in diabetic patients [6].

The components of air pollution constitute over 40 toxic substances from natural and human-made sources. Chronic or excessive exposure to ambient pollutants may result in increased mortality or mortality. The relationship between lung cancer and air pollutants, including nitrogen dioxide, has already been presented in prospective studies [7]. Wu et al. [8] revealed the association between nitrogen dioxide exposure and an increased risk of multimorbidity. Epidemiological studies included in Huang et al.’s meta-analysis [9] pointed out the increased cardiovascular mortality related to chronic exposure to nitrogen dioxide independently of other common air components. Nitrogen dioxide (NO_2_) is a common traffic tracer, and the relationship with acute coronary syndrome risk was revealed in Shearston et al.’s analysis [10].

The air pollution-related risk for coronary artery disease is believed to account for increasing the risk of myocardial infarction, arrhythmia, or stroke by up to 5–10% [11]. Recent studies based on epidemiological data have focused on short-term air pollution exposure and its positive association with acute cardiovascular events [12,13]. In their review, Kulik et al. [14] related the risk of stroke incidence and outcomes to either short- or long-term exposure to ambient pollutants. In animal models [15], chronic exposure to air pollutants promoted atherosclerosis development via inflammatory activation. According to Braunwald [16], up to 20% of cardiovascular deaths are claimed to be air pollution-related.

This study aimed to present a possible relationship between chronic exposure to air pollutants (particulate matter with a diameter of 2.5 microns or less (PM2.5), fine particles with maximal diameter of 10 µm (PM10), or nitrogen dioxide (NO) exposure) and long-term survival in diabetic patients after surgical revascularization.

## 2. Patients and Methods

A total of 140 consecutive diabetic patients (115 (82%) males and 25 (18%) females) with a mean age of 65 (60–71) referred for surgical revascularization due to multivessel coronary disease presenting with chronic coronary syndrome were enrolled in the analysis. The exclusion criteria included patients operated on in an unplanned manner, requiring combined procedures, or patients operated on using cardiopulmonary bypass. This study did not include patients with severe left ventricular dysfunction or an oncological history. The detailed characteristics are presented in Table 1.

### 2.1. Statistical Analysis

The normality of the distribution of variables was tested with the Shapiro–Wilk test. The *t*-test, Cochran–Cox test, Mann–Whitney test, and Fisher’s exact test were used where applicable to compare the variables between the two groups. Logistic regression was performed to analyze the predictors of long-term mortality. Statistical analysis was performed using Statistica 13 by TIBCO. *p* < 0.05 was considered statistically significant.

#### Air Pollution Exposure Personalized Analysis

The basis for assessing the level of individual exposure to air pollution including particulate matter (PM) with diameters of ≤2.5 (PM2.5) and ≤10 µm (PM10) and nitrogen dioxide (NO_2_) for each of the patients was the spatial distribution of air concentration fields for Poland provided by the Chief Inspectorate of Environmental Protection [17]. The maps were based on the results of the national air quality modeling system elaborated by the Institute of Environmental Protection—National Research Institute in Poland (IEP-NRI) based on the legal obligation set out in the Environmental Protection Act in Poland (Art 66, paragraph 6).

### 2.2. Bioethics Committee

Informed consent was obtained from all participants. This study was conducted by the Declaration of Helsinki and approved by the Institutional Review Board (or Ethics Committee) of Poznan University of Medical Sciences, Poznan, Poland (protocol code 55/20 from 16 January 2020) for studies involving humans.

## 3. Results

A total of 140 diabetic patients were operated on using the off-pump technique due to multivessel coronary disease. No perioperative death were noted, and the overall mortality rate was 21 (15%) within 5.5 (5.0–6.1) years of follow-up. The number of grafts performed was 2.4 (0.8), reaching a 86% rate of complete revascularization of 86%. The detailed information is presented in Table 2.

### 3.1. Air Pollution Exposure

The chronic exposure to air pollutants was individually calculated for each patient enrolled in the analysis. The mean values of PM2.5, PM10, and nitrogen dioxide were analyzed. The detailed information is presented in Table 3.

### 3.2. Regression Model

The uni- and multivariable models were created for all-cause mortality risk prediction in the 5-year follow-up, as presented in Table 4.

## 4. Discussion

Our analysis demonstrates the predictive value of air pollutants on all-cause mortality in diabetic patients requiring surgical revascularization. We observed the negative impact of chronic exposure to air nitric oxide concentration above 15 µg/m^3^ on diabetic patients’ survival. In the multivariable model, we also pointed out the protective effect of the completeness of revascularization on 5-year survival.

Diabetes mellitus is a metabolic disorder that implicates inflammatory activation and lipid disturbances in metabolism, causing atherosclerosis formation and propagation. This group of patients is reported to be at higher risk for complications and inferior long-term results, while interventional procedures are proposed.

The multivessel disease represents the advanced stage of coronary atherosclerosis, which can be treated via percutaneous or surgical revascularization to achieve survival benefits. Our multivariable model for 5-year mortality prediction revealed the protective effect of revascularization completeness on patient outcomes. The benefit of complete revascularization (CR) was presented in numerous studies demonstrating its association with mortality reduction [18]. As the minimalization of residual ischemia is the primary target of surgical revascularization, the priority in decision-making was based on the extent of revascularization. The completeness of revascularization can be regarded in the anatomical or viability-guided spectrum [19]. The Syntax Extended Survival Study confirmed the primary role of revascularization completeness achieved by percutaneous or surgical techniques [20]. In Li et al.’s [21] meta-analysis, complete revascularization in acute coronary syndromes significantly reduced the risk for future adverse events compared to culprit-only strategies.

A previous study by Alexeeff et al. [22] suggested the relationship between traffic-related air pollution and increased cardiovascular risk even within neighborhood differences. Boogaard et al.’s [22] meta-analysis demonstrated a moderate association between air pollutants and adverse health outcomes, including increased risk for ischemic heart disease and lung cancer mortality, followed by acute respiratory tract and chronic morbidities. The pathophysiological basis of chronic exposure to air pollutants is linked to enhanced calcium deposition in the vasculature, leading to inflammatory reactions followed by endothelial dysfunction, leading to coronary artery calcification (CAC) [23].

The utility of nitrogen dioxide as a potential coronary artery disease progression factor was presented in our previous analysis [24]. Although air pollutants enter the airways directly, their damaging effect involves other organs, primarily caused by oxidative mediators and inflammatory activations. Among patients exposed to an increased risk of air pollution-induced cardiovascular morbidities, crowded city inhabitants, elderly patients, and those with asthma, chronic obstructive pulmonary disease, and diabetes mellitus are mentioned [25]. The unequivocal evidence of the causative role of air pollutants in cardiovascular diseases has presented the necessity of personalized approaches to minimize devastating health effects [26].

### Study Limitation

This retrospective analysis presents all causes of mortality in diabetic patients operated on due to chronic coronary syndrome using the off-pump technique.

## 5. Conclusions

Chronic exposure to air pollution and arterial revascularization can be regarded as possible long-term survival factors in diabetic patients. Ambient air exposure to nitrogen dioxide above 15 µg/m^3^ may increase the 5-year mortality risk in diabetic patients following surgical revascularization. The personalized approach to optimize long-term results, including the interplay between ambient air pollution and certain co-morbidities, should be considered after surgical revascularization.

## Figures and Tables

**Table 1:** Demographical and clinical characteristics of diabetic groups.

Parameters	Whole Group n = 140	Group 1 Survival Group n = 119	Group 2 Deaths n = 21	*p* Group 1 vs. 2
Follow up time (years) (median, Q1–Q3)	5.6 (5.0–6.1)	5.7 (5.3–6.2)	5.0 (3.7–5.1)	<0.001 *
Demographical				
Age (years) (median, Q1–Q3)	65 (60–71)	64 (59–70)	68 (64–74)	0.072
Sex (n, %)	115 (82)/25 (18)	96 (81)/23 (19)	19 (91)/2 (9)	0.283
BMI (median, Q1–Q3)	29 (27–31)	28 (27–31)	29 (27–32)	0.988
BMI > 30 (n, %)	52 (37)	44 (37)	8 (38)	0.925
Clinical				
Arterial hypertension (n, %)	122 (87)	101 (85)	21 (100)	0.075
Dyslipidemia (n, %)	58 (41)	50 (42)	8 (38)	0.740
Peripheral artery disease (n, %)	17 (12)	12 (57)	5 (24)	0.078
Diabetes mellitus				
on insulin therapy (n, %)	34 (24)	28 (24)	6 (29)	0.591
Non-insulin-dependent (n, %)	106 (76)	91 (77)	15 (71)	0.591
Preoperative laboratory results:				
WBC (10^9^/L) (median, Q1–Q3)	7.55 (6.58–8.75)	7.52 (6.57–8.78)	7.74 (6.83–8.29)	0.979
Neutrophils (10^9^/L) (median, Q1–Q3)	4.94 (4.03–6.24	4.88 (4.03–6.25)	5.26 (4.23–5.92)	0.550
Lymphocyte (10^9^/L) (median, Q1–Q3)	1.89 (1.49–2.24)	1.89 (1.49–2.28)	1.80 (1.45–2.01)	0.295
Hemoglobin (mmol/L) (median, Q1–Q3)	8.6 (8.1–9.2)	8.7 (8.1–9.2)	8.2 (7.5–8.6)	0.101
Platelets (10^3^/L) (median, Q1–Q3)	221 (197–273)	225 (199–273)	213 (193–262)	0.424
Creatinine (µmol/L) (median, Q1–Q3)	92 (79–105)	90.9 (78.5–104)	99.5 (81.0–124.1)	0.130
Uric acid (mg/dL) (median, Q1–Q3)	6.17 (4.86–7.33)	6.12 (4.86–7.30)	6.55 (5.26–8.21)	0.469
Hb1Ac (%) (median, Q1–Q3)	6.76 (6.31–7.01)	6.78 (6.41–7.03)	6.62 (6.22–7.00)	0.178
Preoperative echocardiographic parameters:				
LVED (mm) (median, Q1–Q3)	48 (45–52)	48 (44–51)	50 (47–55)	0.089
IVS (mm) (median, Q1–Q3)	12 (11–13)	12 (11–13)	12 (11–13)	0.511
LVEF (%) (median, Q1–Q3)	58 (50–70)	60 (50–60)	55 (50–60)	0.589
MR (grade) (mean, SD)	1.7 (1.4–1.7)	1.7 (1.5–1.9)	1.5 (1.3–1.8)	0.721

Abbreviations: BMI—body mass index; Hb1Ac—glycated hemoglobin; IVS—intraventrocular septum; LVED—left ventricular end-diastolic diameter; LVEF—left ventricular ejection fraction; MR—mitral regurgitation; Q—quartile; WBC—white blood count; n—number. * Statistically significant.

**Table 2:** Perioperative characteristics of diabetic groups.

Parameters	Whole Group n = 140	Group 1 Survival Group n = 119	Group 2 Deaths n = 21	*p* Group 1 vs. 2
Surgical characteristics				
Number of grafts	2.4 (0.8)	2.4 (0.8)	2.3 (0.9)	0.393
Surgery time	124 (89)/16 (11)	107 (90)/12 (10)	17 (81)/4 (19)	0.238
Postoperative laboratory results:				
WBC (10^9^/L) (median, Q1–Q3)	8.33 (7.18–10.01)	8.54 (7.26–10.01)	8.18 (7.01–9.37)	0.620
Neutrophils (10^9^/L) (median, Q1–Q3)	5.18 (4.27–6.46)	5.17 (4.30–6.33)	5.31 (4.26–7.01)	0.764
Lymphocytes (10^9^/L) (median, Q1–Q3)	1.91 (1.57–2.34)	1.93 (1.61–2.36)	1.67 (1.27–2.01)	0.041
Hemoglobin (mmol/L) (median, Q1–Q3)	7.0 (6.58–7.40)	7.0 (6.6–7.5)	6.9 (6.5–7.2)	0.338
Platelets (10^3^/L) (median, Q1–Q3)	269 (228–337)	275 (235–341)	257 (183–278)	0.066
Troponin–I max (10^9^/L) (median, Q1–Q3)	1.78 (0.72–4.17)	1.67 (0.75–3.87)	2.46 (0.63–5.97)	0.429
Preoperative echocardiographic parameters:				
LVED (mm) (median, Q1–Q3)	48 (45–51)	47 (44–51)	50 (47–52)	0.057
IVS (mm) (median, Q1–Q3)	12 (11–13)	12 (11–13)	12 (11–13)	0.632
LVEF (%) (median, Q1–Q3)	55 (50–60)	55 (50–60)	50 (40–60)	0.018 *
LVEF below 50% (n, (%))	34 (24)	25 (21)	9 (43)	0.032 *
MR (grade) (mean, SD)	1.5 (1.3–1.7)	1.5 (1.4–1.7)	1.4 (1.2–1.6)	0.767
Pharmacotherapy:				
B–blocker, n (%)	140 (100)	119 (100)	21 (100)	1.00
Antiplatelets, n (%)	140 (100)	119 (100)	21 (100)	1.00
ACE–I, n (%)	98 (70)	83 (70)	15 (71)	1.00
ARB, n (%)	42 (30)	37 (31)	5 (24)	0.611
Statin, n (%)	140 (100)	119 (100)	21 (100)	1.00
Ezetimibe, n (%)	45 (32)	41 (35)	4 (19)	0.301
Insulin, n (%)	106 (76)	91 (77)	15 (71)	0.591

Abbreviations: ACE-I—angiotensin-converting enzyme inhibitor; ARB—angiotensin II receptor blocker; IVS—interventricular septum; LVED—left ventricular end-diastolic diameter; LVEF—left ventricular ejection fraction; MR—mitral regurgitation; Q-quartile; SD—standard deviation; WBC—white blood count; n—number. * Statistically significant.

**Table 3:** Air pollution exposure in the presented groups.

Parameters	Whole Group n = 140	Group 1 Survival Group n = 119	Group 2 Deaths n = 21	*p* Group 1 vs. 2
Air pollutants				
PM2.5 (median, Q1–Q3)	19.05 (16.83–22.43)	19.19 (16.83–22.19)	18.52 (16..75–23.93)	0.986
>15 [µg/m^3^] (n, (%))	122 (87)	104 (87)	18 (86)	0.837
>20 [µg/m^3^] (n, (%))	61 (44)	53 (45)	8 (38)	0.587
PM10 (median, Q1–Q3)	25.88 (23.21–29.02)	25.53 (22.59–28.92)	26.24 (23.97–29.19)	0.402
>20 [µg/m^3^] (n, (%))	129 (92)	108 (91)	21 (100)	
>30 [µg/m^3^] (n, (%))	23 (16)	21 (18)	2 (10)	0.358
NO_2_ (median, Q1–Q3)	12.84 (10.59–15.95)	1.67 (0.75–3.87)	2.46 (0.63–5.97)	0.054
>10 [µg/m^3^] (n, (%))	115 (82)	94 (79)	21 (100)	0.014 *
>15 [µg/m^3^] (n, (%))	37 (26)	27 (23)	10 (48)	0.029 *

Abbreviations: NO_2_—nitrogen dioxide; PM 2.5—particulate matter < 2.5 µm in diameter; PM10—particulate matter 2.5–10 µm in diameter; Q—quartile, n—numbers. * Statistically significant.

**Table 4:** Uni- and multivariable models for mortality prediction in diabetic patients.

	Univariable			Multivariable		
	HR	95% CI	*p*	HR	95% CI	*p*
Demographical						
Age	1.06	0.99–1.13	0.088			
Sex (male)	2.28	0.50–10.47	0.291			
BMI > 30	1.05	0.40–2.73	0.922			
Clinical						
Arterial hypertension	2.41	0.95–4.32	0.991			
Dyslipidemia	1.14	0.33–2.20	0.722			
Peripheral artery disease	2.79	0.87–8.96	0.086			
Laboratory						
Creatinine	1.00	0.99–1.01	0.504			
Uric acid	1.13	0.98–1.44	0.311			
Surgical						
Number of grafts	0.78	0.43–1.41	0.405			
Completeness of revascularization	0.48	0.14–1.65	0.242	0.19	0.04–0.86	0.031 *
Echocardiographic						
LV diameter after surgery	1.07	0.99–1.16	0.111			
LVEF < 50% after surgery	2.82	1.07–7.44	0.036 *			
Air pollutants:						
PM2.5 > 20 µg/m^3^	1.31	0.29–1.99	0.584			
PM10 > 30 µg/m^3^	0.49	0.11–2.27	0.363			
NO_2_ > 15 µg/m^3^	2.44	0.93–6.38	0.070	3.99	1.16–13.71	0.028 *

Abbreviations: BMI—body mass index; LV—left ventricular; LVEF—left ventricular ejection fraction; n—number; NO_2_—nitrogen dioxide; PM 2.5—particulate matter < 2.5 µm in diameter; PM10—particulate matter 2.5–10 µm in diameter. * Statistically significant.

## Data Availability

The results of this study will be available from the corresponding authors upon reasonable request for 3 years following the publication.
